# The influence of schooling on working memory performance in elderly individuals without cognitive decline

**DOI:** 10.1590/S1980-57642008DN10300009

**Published:** 2007

**Authors:** Juliana Nery de Souza-Talarico, Paulo Caramelli, Ricardo Nitrini, Eliane Corrêa Chaves

**Affiliations:** 1 MSc, Nursing School Post-Graduation Program, Department of Medical-Surgical Nursing of the University of São Paulo Nursing School and Behavioral and Cognitive Neurology Unit, Department of Neurology, Hospital das Clinicas of the University of São Paulo School Medicine., São Paulo, SP, Brazil.; 2 MD, PhD, Behavioral and Cognitive Neurology Unit, Department of Internal Medicine, Faculty of Medicine of the Federal University of Minas Gerais, Belo Horizonte, MG, Brazil.; 3 MD, PhD, Behavioral and Cognitive Neurology Unit, Department of Neurology and Cognitive Disorders Reference Center (CEREDIC). Hospital das Clinicas of the University of São Paulo School Medicine, São Paulo, SP, Brazil.; 4 PhD, Department of Medical-Surgical Nursing of the University of São Paulo Nursing School, São Paulo, SP, Brazil.

**Keywords:** aging, education, working memory

## Abstract

Over recent decades, research on cognition has been developed rapidly toward better understanding the cognitive changes that usually occur during normal aging. There is evidence that elderly individuals have worse working memory performance than young adults. However, the effect of education on this cognitive function remains unclear.

**Objectives:**

To analyze the performance of healthy elderly subjects on working memory tasks and to verify the influence of educational level on this performance.

**Methods:**

Forty elderly individuals without cognitive impairment and fully independent, were randomly chosen from a group of subjects participating in cultural activities at the university campus. The Digit Span Forward (DSF) test was used to evaluate attention performance. The working memory performance was assessed by the Digit Span Backward (DSB) and the difference between DSF and DSB. The data were statistically analyzed using the Spearman’s correlation coefficient to verify the correlation between the Digit Span (DS) scores and the variables age and schooling, while the Multiple Linear Regression Model was used to verify the effect of these variables on the DS scores.

**Results:**

A significant positive correlation (r=0.41, p<0.01) as well as a significant association (β=0.506; p=0.001; CI 95%= 0.064/0.237) were found between years of schooling and DSB scores. It was not observed statistical correlation (r= –0.08, p=0.64) or association (β=0.41; p=0.775; CI 95%= –0.049/0.065) between age and DSB scores.

**Conclusion:**

In this study, higher levels of schooling were associated with better working memory performance in cognitively healthy elders.

## Abstract (pt)

Ao longo das últimas décadas pesquisas em cognição têm sido desenvolvidas rapidamente na tentativa de compreender as mudanças cognitivas que acontecem durante o processo de envelhecimento. Existem evidências de que indivíduos idosos têm pior desempenho de memória operacional do que adultos jovens. No entanto, o efeito da escolaridade sobre desempenho ainda não foi totalmente esclarecido.

**Objetivos:**

Analisar o desempenho da memória operacional de idosos sem comprometimento cognitivo e verificar a influência da escolaridade neste desempenho.

**Métodos:**

Foram avaliados 40 idosos sem comprometimento cognitivo, independentes, escolhidos aleatoriamente em um grupo de idosos que participaram de uma atividade cultural no campus universitário. Foi utilizado o teste de extensão de dígitos na ordem direta (EDOD) para avaliar a atenção. A memória operacional foi examinada a partir do teste de extensão de dígitos na ordem inversa (EDOI) e a partir da diferença entre EDOD e EDOI. Os dados foram estatisticamente analisados, sendo o Coeficiente de Correlação de Spearman utilizado para verificar correlação entre os escores dos testes de extensão de dígitos (ED) e as variáveis idade e escolaridade e o Modelo de Regressão Linear Múltipla para verificar o efeito destas variáveis nos escores do teste ED.

**Resultados:**

Foi observada correlação positiva significativa (r=0,41, p<0,01) e associação significativa (b=0,506; p=0,001; CI 95%=0,064/0,237) entre escolaridade e o escore do EDOI. Não foi observada correlação (r= –0,08, p=0,64) ou associação ((β=0,041; p=0,775; CI 95%= –0,049/0,065) entre idade e os escores do EDOI.

**Conclusão:**

Neste estudo, maiores níveis de escolaridade associaram-se com melhor desempenho de memória operacional.

Elderly individuals often complain about their memory performance. Over the past few decades, research on cognition has been developed rapidly, toward a better understanding of cognitive changes that usually occur during normal aging. However, the boundaries between normal and pathological aging have yet to be determined.

Differences in memory performance between young adults and healthy elderly subjects are well documented, but not all aspects of memory seem to be equally affected.^1-3^ Elderly subjects exhibit poorer performance in episodic memory, especially on episodic recall or recognition, than young adults.^4,5^ Performance declines during aging for explicit but not implicit tests comparing young and older adults.^6,7^ Semantic memory however, remains relatively unimpaired with ageing, whether tested by explicit or implicit tasks.^8,9^ Increased difficulty with memory for recent events is well known among normal aging,^1,3^ but short-term visual recognition and temporal order memory are both well-preserved in aging.^10^

In addition, there is evidence that older adults have worse working memory performance than young adults.^11,12^ However, the effect of education on test performance remains unclear.^13,14^

Performance on neuropsychological tests is affected by age and education, which makes the early detection of cognitive impairment difficult when assessing individuals with varying levels of education and cultural factors.^15-17^ The influence of education level should be evaluated in any psychometric or neuropsychological assessment of populations with heterogeneous educational background.

Thus, the aims of the present study were to analyze the working memory performance of elderly subjects without cognitive impairment and to verify the influence of educational level on this performance.

## Methods

The present study was carried out at the Cognitive and Behavioral Neurology Unit (CBNU) of the Hospital Clínicas of the University of São Paulo School of Medicine (HC-FMUSP). All data were collected after analysis and approval by the Ethics and Research Committee of the institution, and also by the Ethics Committee of the University of São Paulo School of Nursing. Informed consent was obtained from all participants.

Forty individuals, aged over sixty-years, were included in the study. These individuals were fully independent, with normal cognitive function and randomly chosen from a group of subjects participating in cultural activities at the university campus.

Elderly individuals were excluded if diagnosed with any other neurological or psychiatric disease, or with evidence of cognitive alterations incompatible with the norm for their age, having history of alcohol or drug abuse in the preceding year or for a previous prolonged period, as were individuals using psychoactive medication, as well as illiterate individuals. Cognitive impairment was ruled out based on combination of cognitive and functional evaluation instruments (Mini-Mental State Exam–MMSE^18,19^ and Informant Questionnaire on Cognitive Decline–IQCODE).^20,21^ The MMSE^18^ was employed as a global measure of cognitive function and the following education-adjusted cut-off scores were adopted for the elderly individuals without cognitive impairment: ≥28 for subjects with more than seven years of formal education, ≥24 for subjects with 4 to 7 years, and ≥23 for subjects with 1 to 3 years of schooling.^19^ The IQCODE was employed as a functional evaluation where a cut-off score of ≤3.40 was adopted for individuals without cognitive impairment.^21^ The combination of these two screening tools can increase the diagnostic accuracy of dementia.^21^

All individuals participating in the study were submitted to the study protocol evaluation, which included demographic data (gender, age and schooling) and the Digit Span^22,23^ forward (DSF) and backward (DSB) tests.

The Digit Span test^22,23^ in the Wechsler batteries (the intelligence and memory scales) is the format most commonly used for measuring span of immediate verbal recall. In these batteries it comprises two different tests, the Digit Span Forward (DSF) and Digit Span Backward (DSB) tests, each involving different mental activities.^24^ The DSF test is more closely related to efficiency of attention while the DSB test is related to working memory performance.^24^ Both tests consist of six pairs of random number sequences that the examiner reads aloud at the rate of one per second. In the DSF the subject has to repeat each sequence of number exactly as was given, whereas in the DSB the subject has to repeat each sequence of numbers in exactly the reverse order.^24^ When a sequence is repeated correctly, the examiner reads the next, longer number sequence, continuing until the subject fails a pair of sequences or repeats the highest sequence correctly.^24^ For each digit repeated correctly the subject scores one point on both DSF and DSB for which the maximum score is seven.

All evaluation instruments were administered by the same researcher (JNST) through individual interviews with the elderly subjects.

### Statistical analysis

Initially, all variables were analyzed from a descriptive viewpoint, with determination of means, standard deviations, and minimum/maximum values of the quantitative data (age, schooling, digit span forward and backward scores).

For the categorical variable gender, relative and absolute frequencies were calculated. Non-parametric tests were used because the variables did not present normal distribution. The Mann-Whitney test^25^ was used to compare two independent groups (male and female), whereas Spearman’s correlation coefficient^25^ was used to study the correlation between DS test scores and the variables age and schooling, where the level of significance used for the test was 5% (p<0.05, 95% confidence interval). The independent variables (age and schooling) were input to a multivariate linear regression model for each dependent variable (DSF, DSB, difference of DSF and DSB scores). The independent variables were incorporated into this model through the increase of determination coefficient (R_2_) e p<0.05.

## Results

The group comprised 40 elderly individuals, predominantly female (N=34; 85.0%). Although the female gender predominated there was no statistical difference in demographic data (age and years of schooling) and digit span scores (DSF and DSB) distribution between male and female genders (0.08 ≤ p ≤ 0.93; Mann-Whitney test). Demographic data and Digit Span scores of elderly individuals are shown in Table 1.

**Table 1:** Demographic data, Digit Span forward (DSF) and backward (DSB) scores, MMSE scores and IQCODE scores of elderly subjects.

	Elderly individuals (N=40)		
	Minimum	Mean (SD)	Maximum
Age	62	72.0 (6.3)	90
Years of schooling	1	6.1 (4.2)	19
DSF	5	6.4 (0.7)	07
DSB	2	3.8 (1.3)	07
DSF - DSB	0	2.6 (1.2)	05
MMSE	25	27.1 (1.8)	30
IQCODE	1.6	2.9 (0.4)	3.4

Table 2 shows correlations between digit span scores and demographic data. A positive statistical correlation was observed between DSB score and years of schooling. Therefore individuals with higher schooling presented better performance on the DSB test.

**Table 2:** Correlation between demographic data and Digit Span forward (DSF) and backward (DSB) scores and of elderly subjects.

	Age r (p value)	Years of schooling r (p value)	DSF r (p value)	DSB r (p value)	Difference in DSF and DSB r (p value)
Age	1.00 (-)	-0.11 (0.49)	-0.96 (0.56)	-0.08 (0.64)	-0.00 (0.95)
Years of schooling	-0.11 (0.49)	1.00 (-)	0.28 (0.09)	0.41*(0.00)	-0.25 (0.13)
DSF	-0.01 (0.56)	0.28 (0.09)	1.00 (-)	0.42*(0.00)	0.18 (0.25)
DSB	-0.08 (0.64)	0.41*(0.00)	0.42* (0.00)	1.00 (-)	-0.78* (0.00)
DSF - DSB	-0.00 (0.95)	-0.25 (0.13)	0.18 (0.25)	-0.78*(0.00)	1.00 (-)

* Correlation is significant at the 0.01 level (2-tailed).

Table 3 shows the independent variables (age and years of schooling), their coefficient, confident intervals and p values obtained from the multiple linear regression analysis model in relation to the dependent variables (DSF, DSB and DSF-DSB). The variable “years of schooling” was significantly associated with DSB and DSF-DSB. Moreover, the regression model proposed for the dependent variables indicates that for each year of schooling the DSB score increases 0.506 units while DSF-DSB decreases 0.349 units.

**Table 3:** Independent variables, β coefficient, β confidence interval and p values obtained using the model of multiple linear regression in relation to dependent variable Digit Span forward (DSF), backward (DSB) scores and the difference between DSF and DSB (DSF-DSB) scores in elderly subjects.

Dependent variable	Independent variable	Beta (β)	p value	CI _95%_β [min./max.]
	Intercept	6.019	0.000	[3.345/8.692]
DSF	Years of schooling	0.228	0.168	[-0.017/0.095]
	Age	-0.098	0.549	[-0.048/0.026]
	Intercept	1.151	0.568	[-2.899/5.201]
DSB	Years of schooling	0.506	0.001	[0.064/0.237]
	Age	0.041	0.775	[-0.049/0.065]
	Intercept	4.868	0.017	[0.921/8.814]
DSF - DSB	Years of schooling	-0.349	0.030	[-0.184/-0.010]
	Age	-0.140	0.371	[-0.083/0.032]

## Discussion

The results from the present study show that schooling is associated with working memory performance. Therefore, elderly individuals with higher education level have better working performance than those with low education level. Moreover, low differences between the forward and backward performance are expected in elderly individuals with higher education level.

Moreover, it is important to observe that scores of 4 or 5 on the DSB spans are within normal limits, while a span of 3 may be borderline or defective.^24^ However, education level appears to have a decisive effect on this task^26,27^ and for some tests, only 1 or 2 years of formal education may result in a significant difference in test performance.^28^ In the present study a subject with one year of schooling scored 2 points in this test, but this lower score was not associated with cognitive impairment, which was evaluated through the association between MMSE and IQCODE.

The educational effect on neuropsychological test performance, included in the Digit Backward test, is not a linear effect. Differences between 0 (illiterate) and 3 years of education are usually highly significant, whereas no differences are expected to be found between, for example, 12 and 15 years of education.^28^

Several proposals have been presented to explain this effect of education frequently found for at least some tests of neuropsychological functioning. One proposal is related to the socioeconomic status and its effect on brain reserve capacity in early life.^29^ Another explanation for the effect of education on neuropsychological performance proposes that education level increases brain reserve by increasing synaptic density in neocortical association cortex.^30^ This hypothesis is supported by the fact that increased synaptic density is expected in highly-educated people.^30^ This increased brain reserve may delay the onset of dementia by some 4 to 5 years.^30^

Some previous studies,^31-34^ but not all,^35,36^ have found an association between education level and working memory performance. In one of these studies, for example, a correlation was found between schooling and both DSF and DSB.^33^ However, the mean schooling of this sample was lower in comparison with our study. These results suggest that it would be interesting to analyze working memory performance in elderly individuals distributed in different clusters of schooling, to evaluate which levels are more critical to memory performance.

According to the working memory framework^37,38^ this model of memory has four components: the central executive, the visuospatial sketchpad, the phonological loop and the episodic buffer, which is assumed to form a temporary storage system that allows information from the subsystems to be combined with that from long-term memory into integrated chunks. This model provides basis for tackling the more complex aspects of executive control in working memory.^37,38^ The DSB test involves mental double-tracking in that both the memory and the reversing operations must proceed simultaneously.^24^ Many people report that they perform this task by making a mental image of the numbers and “reading” them backwards.^24^ This mental image of the numbers is related to the visuospatial sketchpad component of the working memory model. This subsystem of working memory serves the function of integrating spatial, visual, and possibly kinesthetic information into a unified representation which may be temporarily stored and manipulated.^38^ Thus, perhaps the system’s capacity to manipulate and bind information from the subsidiary systems and long-term memory in individuals with higher educational level, works in a more effective way than in individuals with lower educational level.

Moreover, there is evidence that elderly individuals have compensatory mechanisms, such as additional prefrontal cortical activity, to maintain proficiency on working memory performance. However, when the cognitive demand increases they are pushed past a threshold beyond which physiological compensation cannot be made and decline in performance occurs.^39^ Thus, it is possible that elderly individuals with higher educational level have more ability to maintain better performance than individuals with lower educational level.

In addition, in our study the worse performance on elderly individuals with low education can be related to processing speed. A previous study has found that the processing speed in working memory was worse in less-educated individual than in well-educated participants.^40^

Though the association we observed was statistically significant in a multiple linear regression model, it is important to consider that few independent variables were used. Therefore, other variables, such as long term memory and executive functions and other potential confounders. In addition, the sample was generally small and may not be clinically meaningful. A study with elderly in different clusters of schooling, with higher number of individuals and a variety of ethnic groups and socioeconomic backgrounds is warranted, since it may show more consistent results. Moreover, in relation to working memory, we used just one test. Other cognitive instruments should be applied to evaluate more accurately the working memory performance.

Our findings emphasize the influence of schooling on working memory performance. These results indicate that neuropsychological assessment must consider the effects of educational level on working memory performance in the early detection of cognitive impairment, especially in countries with heterogeneous educational background. It is necessary to establish cut-off scores according to education in elderly individuals to make an accurate diagnosis of cognitive impairment and dementia. Despite its limitations, the findings of the present study are very consistent with the hypothesis that educational level influences working memory performance in elderly individuals.
